# The Brompton Breathing Pattern Assessment Tool (BPAT): A Journey to Identify the Unknown, a Decade on…

**DOI:** 10.1002/resp.70245

**Published:** 2026-03-26

**Authors:** James H. Hull, Lizzie Grillo

**Affiliations:** ^1^ Royal Brompton Hospital, Guys and St Thomas' NHS Foundation Trust London UK; ^2^ Institute of Sport, Exercise and Health (ISEH), UCL London UK; ^3^ National Heart and Lung Institute (NHLI) Imperial College London London UK

**Keywords:** assessment, asthma, breathing pattern disorder, breathlessness, dysfunctional breathing

It is almost a decade since a novel clinical instrument, the Brompton Breathing Pattern Assessment Tool (BPAT), was first described in this journal [1]. The BPAT was developed to help clinicians detect and characterise breathing patterns and to support diagnosis of under‐identified breathlessness, termed dysfunctional breathing or a breathing pattern disorder (BrPD).

The concept of BrPD has evolved considerably over time. Historically described as ‘hyperventilation syndrome’, the condition was largely framed as psychologically driven, associated with anxiety and panic. Assessment focused on provoking hyperventilation and measuring physiological responses [2]. Subsequently, the broader term ‘dysfunctional breathing’ emerged, acknowledging that abnormal breathing may involve altered biomechanics, heightened breathing awareness and behavioural adaptations, rather than hyperventilation alone [3]. This reframing moved the condition beyond a psychiatric construct, highlighting its clinical relevance in respiratory disease, where a disordered breathing pattern may amplify symptom burden [1, 4, 5].

More recently, consensus statements have endorsed the abbreviation BrPD, to improve conceptual clarity and reduce confusion arising from interchangeable terminology [6]. Multiple classification frameworks have been proposed, including those proposing a distinction between thoracic and extra‐thoracic forms of the condition, descriptions of specific biomechanical patterns, for example, thoracic‐dominant breathing or thoraco‐abdominal asynchrony [7], and multidimensional models incorporating biochemical, biomechanical, and psychophysiological domains [3].

Despite improved recognition of BrPD, there remains no gold standard diagnostic criterion or single instrument capable of capturing the multidimensional nature of this condition. Consequently, BrPD continues to be under‐recognised, and awareness of appropriate screening and referral pathways remains variable among clinicians [3, 6].

The BPAT was developed within our specialist asthma service to improve identification of BrPD. Individuals completed a structured multidisciplinary assessment, allowing concurrent investigation to explore factors contributing to refractory disease. A substantial proportion of patients demonstrated disproportionate breathlessness, often accompanied by observable breathing pattern abnormalities (Table 1).

**TABLE 1:** BPAT studies.

Study	Year	Journal	Population	Key findings	References
Todd et al. [1]	2018	Respirology	Treatment‐refractory asthma (*n* = 150)	BPAT detected BrPD with sensitivity 0.92, specificity 0.75. BrPD is highly prevalent in refractory asthma.	Respirology 2018;23:284–90
Bondarenko et al. [8]	2021	Clinical & Experimental Allergy	Stable asthma (*n* = 30)	High inter‐rater reliability for BPAT in both in‐person and remote (video) assessment.	Clin Exp Allergy 2021;51:1218–20
Hylton et al. [9]	2022	Clinical Medicine	Post‐COVID breathlessness (*n* = 19)	BPAT sensitivity 89.5%, specificity 78.3% for diagnosing BRPD in long COVID patients.	Clin Med 2022;22:376–79
Reilly et al. [10]	2023	Autonomic Neuroscience	POTS with dysfunctional breathing (*n* = 77)	BPAT sensitivity 87%, specificity 75% for identifying DB in POTS (AUC 0.901).	Auton Neurosci 2023;248:103104
Vilarinho et al. [11]	2024	Pulmonology	Translation/adaptation study	Portuguese translation and cultural adaptation of BPAT completed and validated.	Pulmonology 2024;30:646–47
Sedeh et al. [12]	2020	Respiratory Medicine	*N* = 117 with ‘difficult asthma’	Patients with objective signs of DB according to the BPAT score had worse asthma control: BPAT > 4 vs. < 4: (ACQ: Mean (SD) 3.15–0.93 vs. 2.03–1.15).	Respir Med. 2020;163:105894.
Freitas et al. [13]	2026	Physiotherapy Research international	*N* = 18 adults with COPD and 4 Assessors	Reliability of 4 assessors (ICC 0.70 [0.50–0.86]) and 3 remote assessors (ICC 0.70 [0.47–0.86]). Bland–Altman analyses ranged from −1.17 to −0.22 points.	Physiother Res Int. 2026;31(1):e70124.

This presentation was increasingly recognised within other asthma populations, with services utilising the Nijmegen Questionnaire (NQ) to screen for hyperventilation‐related symptoms [4]. However, there were concerns that the NQ may not recognise or detect BrPD and potentially misattribute asthma‐related symptoms to hyperventilation [5]. We observed that questionnaire‐based screening was suboptimal when compared with a more detailed assessment undertaken by a specialist respiratory physiotherapist, highlighting the need for a structured, pragmatic observational tool.

Our objective was to develop an instrument that could be applied by clinicians with varying experience and avoiding tactile assessment of the thoracic cage, which may alter breathing mechanics. We developed the BPAT through structured discussion of the core physiotherapy assessment components. It evaluates seven domains: (i) chest and abdominal wall movement; (ii) inspiratory flow characteristics; (iii) expiratory flow characteristics; (iv) route of inspiration and expiration; (v) observable signs of air hunger; (vi) respiratory rate; and (vii) rhythm. Each domain is scored from 0 to 2 according to deviation from expected normal features, yielding a total score ranging from 0 to 14. Assessment is performed over 1 min with the patient seated comfortably and supported, following at least 5 min of quiet rest.

The BPAT is designed as a formative measure, with individual items capturing distinct, observable features of breathing, which together describe a patient's breathing pattern. Unlike reflective instruments, the items are not assumed to reflect a single underlying trait or dimension; instead, each item contributes information to the overall clinical picture.

Patients with physiotherapist‐diagnosed BrPD, with or without asthma, demonstrated significantly higher BPAT scores than those without BrPD [1]. Receiver operating characteristic analysis demonstrated good discrimination (AUC 0.88), with a cut‐off ≥ 4 yielding sensitivity of 0.92 and specificity of 0.75. The BPAT correlated poorly with symptom questionnaires but more strongly with respiratory rate. As with all tools in this area, precision is limited by the absence of an independent gold standard reference. This foundational study provided encouraging preliminary evidence supporting the BPAT's role in structured clinical assessment.

Consequently, the BPAT has been adopted internationally, including a Portuguese translation [11], utilized in other asthma cohorts [12], long COVID [9], COPD [13] and in people with postural orthostatic tachycardia syndrome [10]. Higher BPAT scores are associated with greater breathlessness and hyperventilation symptoms, and poorer asthma control [12]. In asthma, the BPAT demonstrates excellent inter‐rater reliability for in‐person assessment between physiotherapists (ICC 0.95, 95% CI 0.91–0.98) and good reliability when conducted via video assessment (ICC 0.76, 95% CI 0.56–0.88) in asthma [8] and in COPD (ICC 0.70, 95% CI 0.47–0.86) [13]. Reduced reliability in remote assessment appears attributable to limitations in audio clarity.

Although the BPAT demonstrates promising clinical utility, further evidence is required of its clinometric properties, including responsiveness and minimally important difference. One limitation is also that it evaluates observable resting breathing features and does not fully encompass the multidimensional nature of BrPD, which involves interacting symptom burden, altered interoceptive experience, and variable physiological findings.

The BPAT captures a single timepoint measurement of an individual's *resting* breathing pattern. However, in this population, exertion frequently provokes symptoms with variability in daily symptoms. Longitudinal tools, both at rest and during exercise, are also required. Advances in characterising the different forms of BrPD have arisen through cardiopulmonary exercise testing (CPET) [14] and optoelectronic plethysmography [15]. CPET offers particular value by excluding significant cardiac or pulmonary pathology whilst simultaneously identifying abnormal ventilatory control patterns. This supports a shift away from viewing BrPD as a diagnosis of exclusion toward one informed by measurable physiological features.

While we feel proud to have developed a tool that has helped many become aware of BrPD in their clinical populations, it remains a challenging and elusive condition to evaluate. The BPAT helps characterise the biomechanical component of breathing pattern assessment, supporting a positive clinical diagnosis of BrPD when combined with symptom screening and optimisation of co‐morbid conditions. However, further work is needed to develop improved diagnostic tools in this clinically important area and to support the evolution of the BPAT over the coming decade.

## Funding

The authors have nothing to report.

## Conflicts of Interest

Copyright of the BPAT resides with Imperial College London, Innovahealthtech (https://www.innovahealthtec.com/BPAT). The BPAT is free to use for healthcare organisations for non‐commercial internal business only. A licence is required for use and available via Innovahealthtech website (https://www.innovahealthtec.com/contact‐us). There are no other conflicts of interest.

## Data Availability

Data sharing not applicable to this article as no datasets were generated or analysed during the current study.
